# Misuse of psychotropic medications among hospitalized patients

**DOI:** 10.1192/j.eurpsy.2023.1372

**Published:** 2023-07-19

**Authors:** F. Azraf, A. Tounsi, M. Sabir, F. El Omari

**Affiliations:** Arrazi hospital, Faculty of Medicine and Pharmacy of Rabat, sale, Morocco

## Abstract

**Introduction:**

Psychotropic drugs are sometimes taken outside of any medical context and can be diverted or even trafficked. This phenomenon affects more adolescents and young adults, more specifically those who are already struggling with other consumption problems. This vulnerable population is thus exposed to several negative health and social repercussions.

**Objectives:**

The objective of our work is to determine the sociodemographic characteristics of the patients, the main drugs used, the dose, the route of administration, the mode of obtaining the drug and the intentionality of the use.

**Methods:**

It is a retrospective study spread over 1 year on a series of patients who were hospitalized in the service of addictology at the psychiatric hospital Ar-razi of Salé. The data collection was carried out from the medical files and an exploitation form.

**Results:**

A total of 141 patients were hospitalized during this period of which 53 37.5 had a diverted use of medication. Our sample is characterized by a predominance of female patients (90%) with a median age of 33 years. The most misused drugs were benzodiazepines (85.7%), analgesics (11.7%), hypnotics (9.4%) and antihistamines (1.9%). Misuse is related to exceeding doses (up to 40 times the maximum dose of the AMM) and duration of treatment, to use or obtaining drugs without a prescription 79.2 , to the route of administration and to drug addiction. 11.3 of patients were dependent on a single drug; 88.7% of patients had a co-addiction: tobacco 86.8%, cannabis 77.3%, alcohol 58.5%, cocaine 34.5%, solvents 15 , heroin 1.8% or a history of addiction. Hospitalization was only related to drug misuse in 11.3% of cases. The effects sought: anxiolysis 58.5%, euphoria 30.2%, analgesic 5.7%, disinhibition 3.8%, increased performance 1.9%

**Conclusions:**

The misuse of psychotropic drugs is a well-known public health problem. However, this phenomenon remains unknown and neglected in Morocco.

**Disclosure of Interest:**

None Declared

